# Repetitive transcranial magnetic stimulation restores altered functional connectivity of central poststroke pain model monkeys

**DOI:** 10.1038/s41598-021-85409-w

**Published:** 2021-03-17

**Authors:** Yoshinori Kadono, Keigo Koguchi, Ken-ichi Okada, Koichi Hosomi, Motoki Hiraishi, Takashi Ueguchi, Ikuhiro Kida, Adnan Shah, Guoxiang Liu, Youichi Saitoh

**Affiliations:** 1 Department of Neurosurgery, Graduate School of Medicine, Osaka University, Suita, Japan; 2 Laboratories for Neuroscience, Visual Neuroscience Group, Graduate School of Frontier Biosciences, Osaka University, Suita, Japan; 3 Center for Information and Neural Networks (CiNet), National Institute of Information and Communications Technology, and Osaka University, Suita, Japan; 4 Department of Physiology, Hokkaido University School of Medicine, Sapporo, 060-8638 Japan; 5 Department of Neuromodulation and Neurosurgery, Graduate School of Medicine, Osaka University, Suita, Japan

**Keywords:** Neuroscience, Chronic pain, Stroke, Neuropathic pain

## Abstract

Central poststroke pain (CPSP) develops after a stroke around the somatosensory pathway. CPSP is hypothesized to be caused by maladaptive reorganization between various brain regions. The treatment for CPSP has not been established; however, repetitive transcranial magnetic stimulation (rTMS) to the primary motor cortex has a clinical effect. To verify the functional reorganization hypothesis for CPSP development and rTMS therapeutic mechanism, we longitudinally pursued the structural and functional changes of the brain by using two male CPSP model monkeys (*Macaca fuscata*) developed by unilateral hemorrhage in the ventral posterolateral nucleus of the thalamus. Application of rTMS to the ipsilesional primary motor cortex relieved the induced pain of the model monkeys. A tractography analysis revealed a decrease in the structural connectivity in the ipsilesional thalamocortical tract, and rTMS had no effect on the structural connectivity. A region of interest analysis using resting-state functional magnetic resonance imaging revealed inappropriately strengthened functional connectivity between the ipsilesional mediodorsal nucleus of the thalamus and the amygdala, which are regions associated with emotion and memory, suggesting that this may be the cause of CPSP development. Moreover, rTMS normalizes this strengthened connectivity, which may be a possible therapeutic mechanism of rTMS for CPSP.

## Introduction

Central poststroke pain (CPSP), which is intractable central neuropathic pain that occurs in 1–12% of stroke patients, is typically associated with strokes occurring around the somatosensory pathway including the ventral posterolateral nucleus (VPL) of the thalamus, anterior pulvinar, and lateral medulla^1–4^. The time to CPSP onset after stroke varies between patients, although CPSP usually develops weeks or months after the stroke event^3^. This late-onset characteristic indicates that the pathophysiological background of CPSP includes a progressive, adaptive mechanism that involves maladaptive plastic changes and reorganization of the pain network^5^. A stroke in the lateral somatosensory pathway can lead to CPSP; however, the resulting functional changes would also occur in the medial emotional pathway, which includes the amygdala, anterior cingulate cortex, and insular cortex. Consistent with the network reorganization hypothesis, some invasive neurostimulation treatments such as electrical motor cortex stimulation and deep brain stimulation to the thalamus or brainstem have the therapeutic effect of alleviating pain^6–10^. Noninvasive stimulation of the primary motor cortex (M1) by a high-frequency (5–20 Hz) repetitive transcranial magnetic stimulation (rTMS) also has a therapeutic effect on CPSP^11,12^.

Previous brain imaging studies have reported differences in brain activity between stroke patients with CPSP and without CPSP^13^. However, a causal relationship between such differences and the symptoms has not been established because a direct comparison of the functional and anatomical changes in the human brain before and after a stroke is virtually impossible. A nonhuman primate model of CPSP using the rhesus macaque monkey was recently established by inducing a haemorrhagic lesion in the VPL^14^. The macaque monkey has sufficient anatomical similarity to humans and the model has the late-onset characteristics like human CPSP patients. Thus, the developmental mechanism may reproduce human CPSP. The CPSP model monkey enables scientists to longitudinally pursue the brain activity changes after CPSP development and the therapeutic mechanism of rTMS.

In this study, we created the monkey models of CPSP and administered rTMS to them, while tracking changes in pain threshold by using a behavioural experiment and tracking anatomical and functional changes in the brain by using magnetic resonance imaging (MRI). In particular, to address the network reorganization hypothesis, we aimed to examine the changes in functional connectivity among pain-related brain regions using resting-state functional MRI (rs-fMRI) before and after CPSP development and with and without rTMS for CPSP. Moreover, we compared the results to former clinical research and verified the network reorganization hypothesis.

## Results

### Lesion induction

**Figure 1:**
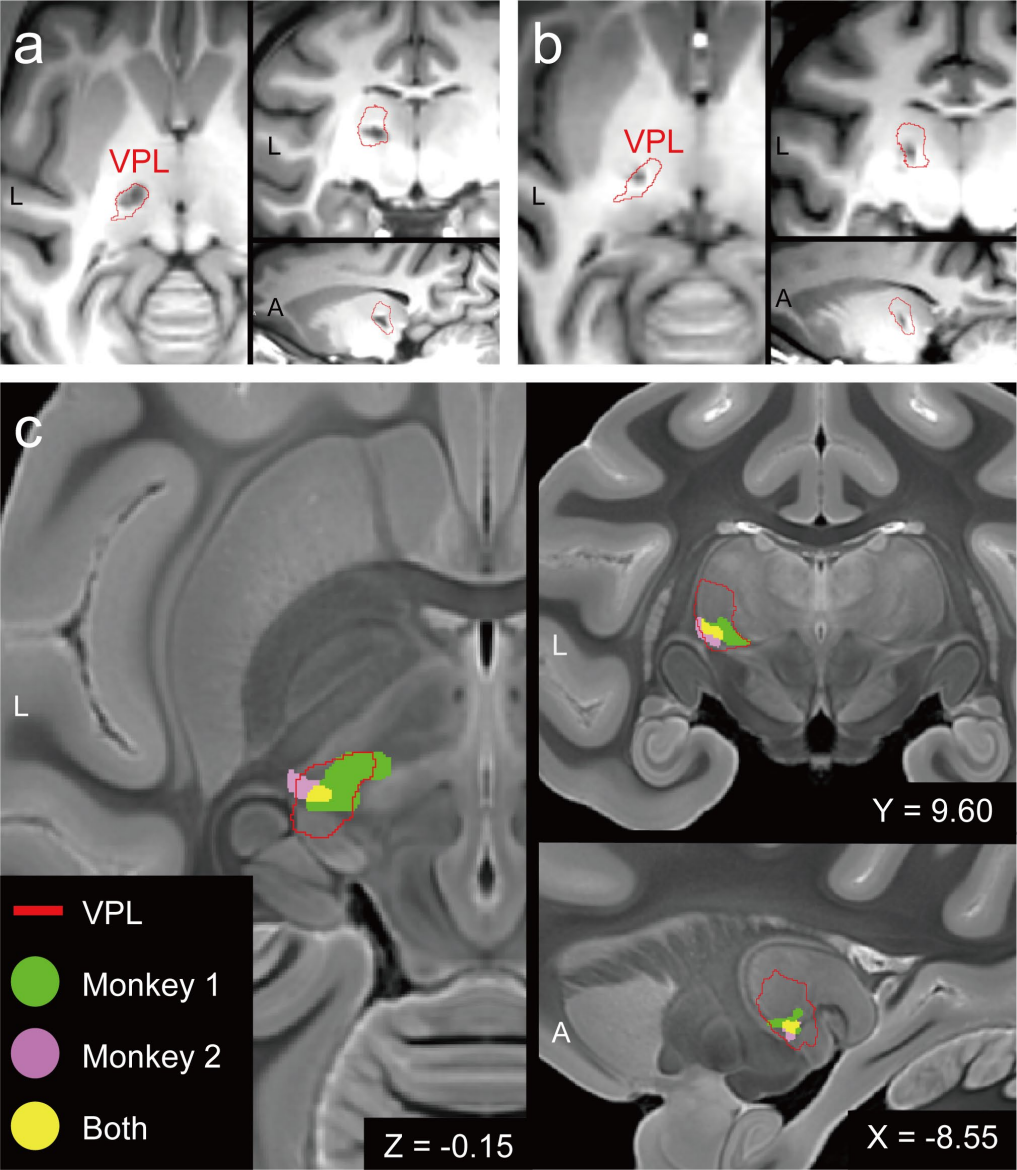
Location of the induced lesion on magnetic resonance images. Axial (left), coronal (upper right) and sagittal (lower right) slices are shown. (**a**,**b**) Individual T1-weighted magnetic resonance images taken 1 month after the collagenase injection for monkey 1 (**a**) and monkey 2 (**b**). The red lines indicate the edge of the VPL for lesion side warped from a monkey atlas. The lesions appear as low-intensity areas within the VPL. (**c**) The warped lesion for the two monkeys overlaid on the T2-weighted template image. Green, pink, and yellow areas indicate the lesion for monkey 1, the lesion for monkey 2, and the overlapped area, respectively. The xyz coordinate is defined at the anterior commissure. *A* anterior, *L* left, *VPL* ventral posterolateral nucleus in the thalamus.

We made two CPSP model monkeys by the stereotactic microinjection of collagenase type IV into the left VPL hand area. The lesion site was checked with structural MRI scans and by using an available macaque atlas^15^. A few days after the injection, vague lesions were expanding for whole VPL areas, accompanied by haematoma and oedema for surrounding structures involving internal capsules adjacent to the VPL (Supplementary Fig. S1). Approximately 1 month after lesion induction (monkey 1: 36 days; monkey 2: 38 days), the lesions had clarified and the oedema had disappeared (Fig. 1). The lesions, which appeared with low intensity on T1-weighted images, were in the VPL as indicated by the atlas for both monkeys (Fig. 1a,b). The lesions of two monkeys overlapped at the centre of the ventral VPL in the template atlas space (Fig. 1c). Slight individual differences in the lesion site existed: for monkey 1, the lesion was in the most ventral centre of VPL and slightly spreading in the direction of the anterior pulvinar nucleus, whereas for monkey 2, the lesion was localized ventrolateral of the VPL contacting the posterior limb of the internal capsule.

### rTMS increased the pain thresholds of the CPSP monkeys

**Figure 2:**
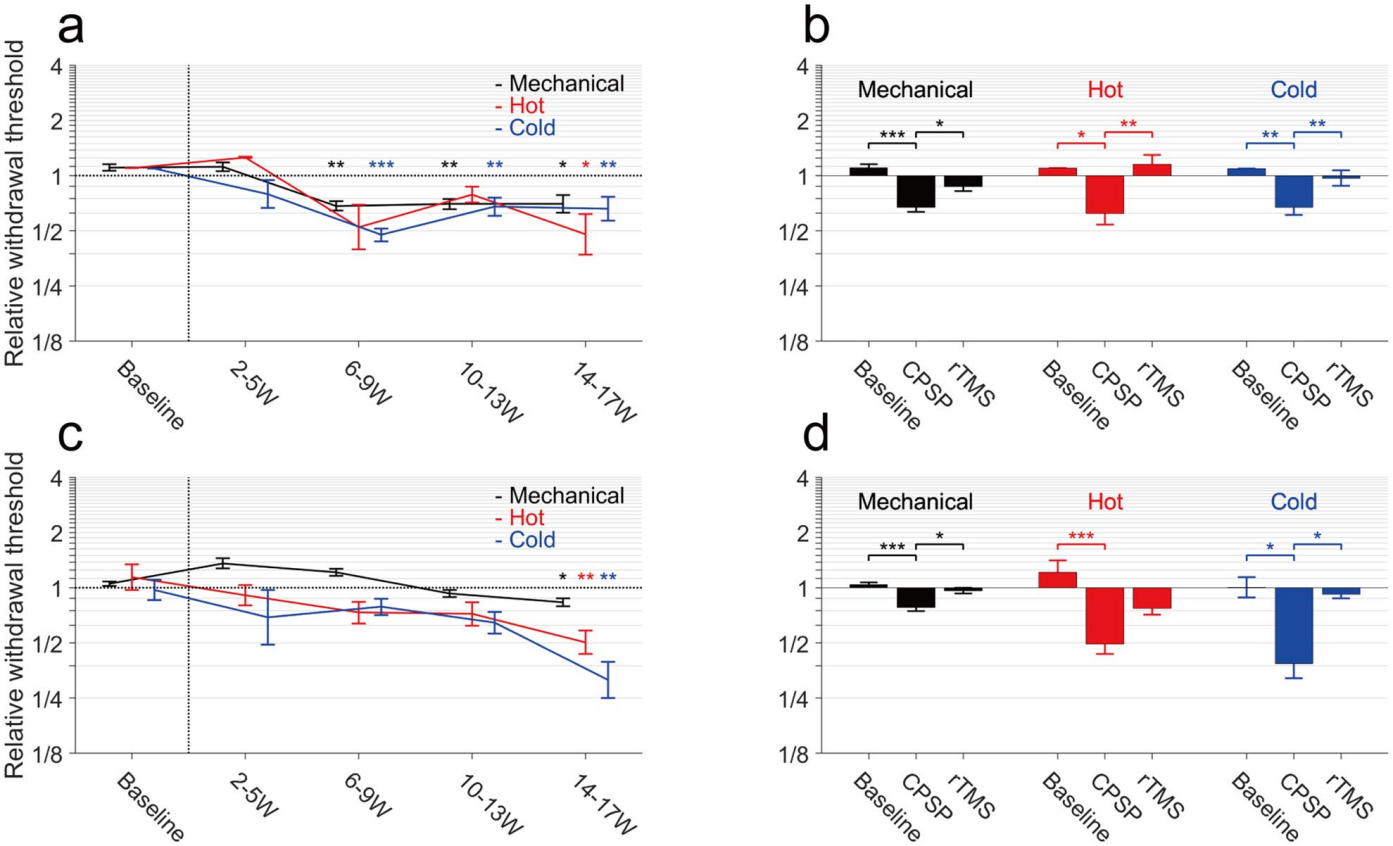
Behavioural changes after lesion and post rTMS treatment. Medians and interquartile ranges for the relative withdrawal threshold of the affected side (contralateral to the lesion) against the unaffected side (ipsilateral to the lesion) are shown. (**a**,**c**) The changes in relative withdrawal latency to mechanical (black) and thermal stimulation (red: hot, blue: cold) are shown before and after lesion induction for monkey 1 (**a**) and monkey 2 (**c**). After 14 weeks following the injection, the relative withdrawal latency for all stimulations significantly decreased relative to those before the injection. (**b**,**d**) Comparison of the withdrawal thresholds for the prelesion baseline, CPSP condition, and after rTMS treatment for monkey 1 (**b**) and monkey 2 (**d**). After the rTMS treatment, the reductions were recovered to the baseline without monkey 2 against hot stimulation. *p < 0.05, **p < 0.01 and ***p < 0.001, based on Kruskal–Wallis test followed by Dunn’s post hoc analysis (**a**,**c**), and based on Wilcoxon signed-rank test (**b**,**d**). *CPSP* central poststroke pain, *rTMS* repetitive transcranial magnetic stimulation.

Figure 2 shows the changes in pain perception in the forelimbs after the VPL lesion. We compared the relative withdrawal threshold of the affected side (i.e., contralateral to the lesion) against the unaffected side (i.e., ipsilateral to the lesion) for mechanical and thermal stimulations. The relative withdrawal threshold of both monkeys tended to decrease gradually after the lesion, regardless of the stimulation modality (Fig. 2a,c), which suggested the development of hyperalgesia.

With the mechanical stimulation of monkey 1, the relative withdrawal threshold 6–9 weeks after injection and later were significantly lower than the threshold at baseline (Fig. 2a, *p* < 0.05, Kruskal–Wallis test followed by Dunn’s post hoc analysis). For monkey 2, the withdrawal threshold initially increased (Fig. 2c), and it seemed to express sensory disturbance and/or motor weakness after the lesion. However, in the later period, the relative withdrawal threshold was significantly lower than the threshold before the VPL lesion (after 14–17 weeks, *p* = 0.028). For the hot temperature stimulation, the withdrawal threshold after 14–17 weeks in both monkeys was significantly lower than the threshold before the lesion (Fig. 2a, monkey 1 at 48 °C, *p* = 0.017; Fig. 2c, monkey 2 at 50 °C, *p* = 0.016). For the cold temperature stimulation, the relative withdrawal threshold of monkey 1 significantly decreased 6–9 weeks after injection and later (Fig. 2a, at 10 °C, *p* < 0.0001). For monkey 2, a relative withdrawal threshold for 10 °C did not significantly decrease even in 14–17 weeks (*p* = 0.32). In an additional experiment using 5 °C stimulation for monkey 2 after the lesion, although the prelesion data were lacking and substituted by that of 10℃, the relative withdrawal threshold in 14–17 weeks was significantly decreased (Fig. 2c, p = 0.0013). Based on these results, the time of the appearance of hyperalgesia was approximately 2 months for monkey 1 and approximately 3 months for monkey 2.

After confirming the development of CPSP, we then investigated whether the rTMS affected the withdrawal threshold of the monkeys (in 14–18 weeks for monkey 1; 18–22 weeks for monkey 2). In the same time period, we also examined the withdrawal threshold with no stimulation and with sham stimulation. No difference existed between the data with sham stimulation and the data with no stimulation. Thus, we treated both data as the CPSP data. Figure 2b,d show the comparison of withdrawal threshold between the prelesion baseline, CPSP condition, and after rTMS. In the CPSP condition, the pain thresholds for both monkeys were decreased compared to baseline. After application of rTMS, the pain threshold in monkey 1 was increased for mechanical, hot, and cold stimulation (Fig. 2b; mechanical, *p* = 0.032; at 48 °C, *p* = 0.005; at 10 °C, *p* = 0.008; Wilcoxon signed-rank test). In monkey 2 (Fig. 2d), the pain thresholds for mechanical and cold stimulation were significantly increased (mechanical, *p* = 0.036; at 5 °C, *p* = 0.014), but the threshold for hot stimuli did not reach the significance level (at 50 °C, *p* = 0.13). Overall, the decreased pain thresholds of CPSP models recovered to the baseline level by rTMS.

### Changes in structural connectivity after VPL lesion

**Figure 3:**
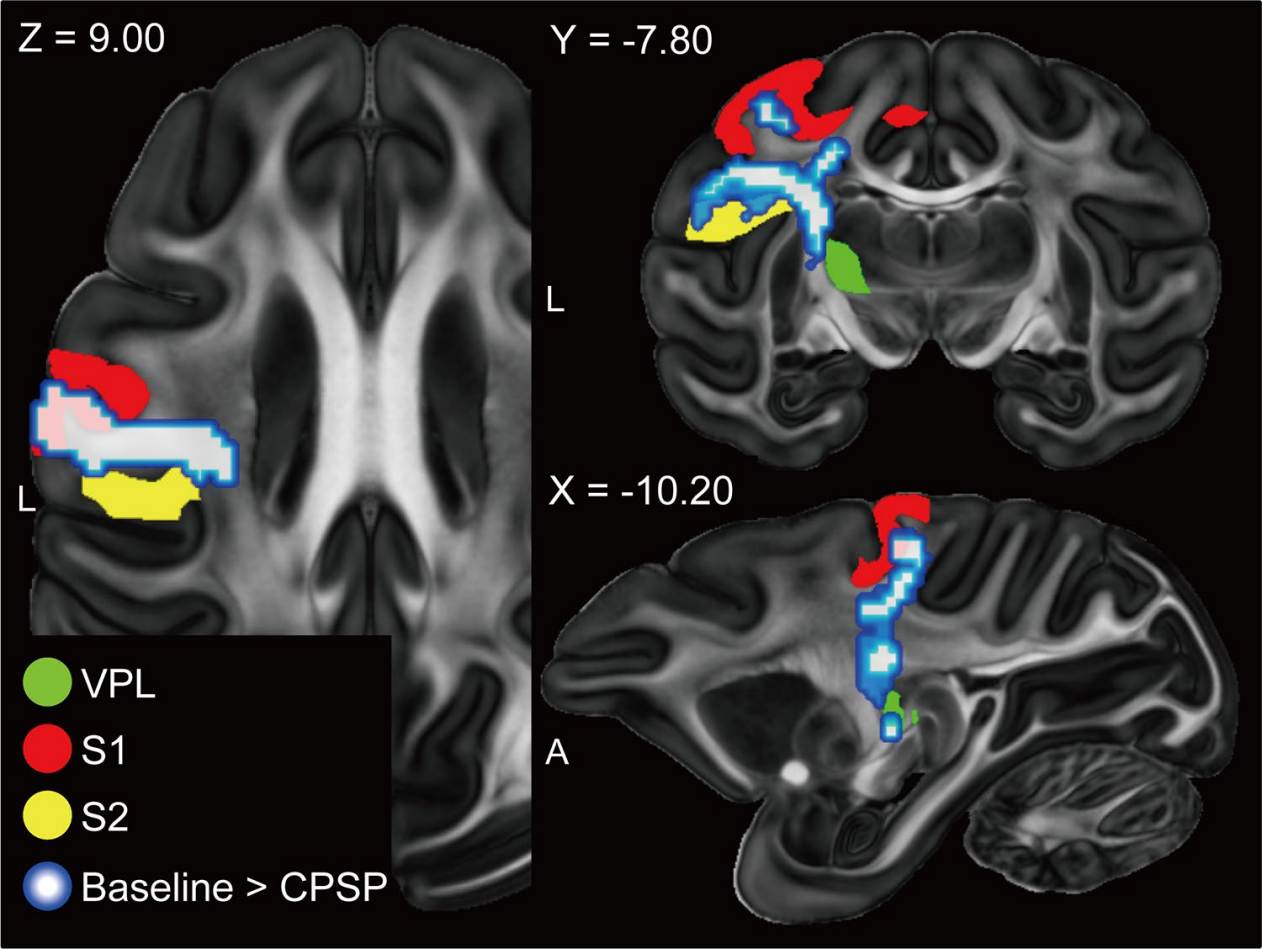
Changes in structural connectivity after CPSP development overlaid on the fractional anisotropy image of the DTI-based atlas. For tractography, the lesion side of the entire thalamus was selected as the seed. The axial (left), coronal (upper right), and sagittal (lower right) slices are shown. The xyz coordinate is defined at the anterior commissure. The blue areas indicate that the structural connectivity was decreased after the lesion (uncorrected *p* < 0.001 and cluster-size adjusted *p*-FDR < 0.05). The red, yellow and green areas are S1, S2 and VPL, respectively, marked from the template atlas. *A* anterior, *CPSP* central poststroke pain, *FDR* false discovery rate, *L* left, *S1* primary somatosensory cortex, *S2* secondary somatosensory cortex, *VPL* ventral posterolateral nucleus in the thalamus.

The anatomical and functional changes in the brain were analysed by collecting MRI data after CPSP development (monkey 1, 14–23 weeks; monkey 2, 15–23 weeks) and compared to that of pre-lesion baseline data. Diffusion-tensor imaging (DTI) data were analysed to detect the induced changes in structural connectivity after the lesion that extended outside the VPL. We tracked the streamlines that passed through the entire thalamus as the index of structural connectivity. On the lesioned side, the structural connectivity was significantly decreased (*p* < 0.001) within the white matter between the VPL and S1/S2 areas after CPSP development (Fig. 3), although the damage was incomplete and streamlines remained (Supplementary Fig. S2). These decreased structural connectivities were on the ascending spinothalamocortical pathways. However, the structural connectivity that passed through the thalamus on the opposite side of the lesion was unchanged. Furthermore, any changes in structural connectivity between CPSP and rTMS conditions could not be found. These results demonstrated that the VPL lesion induced a reduction in the structural connectivity on the ascending spinothalamocortical pathways, and that the rTMS for the CPSP monkeys did not have any effect on structural connectivity.

### Changes in functional connectivity in brain regions after CPSP development and rTMS

**Figure 4:**
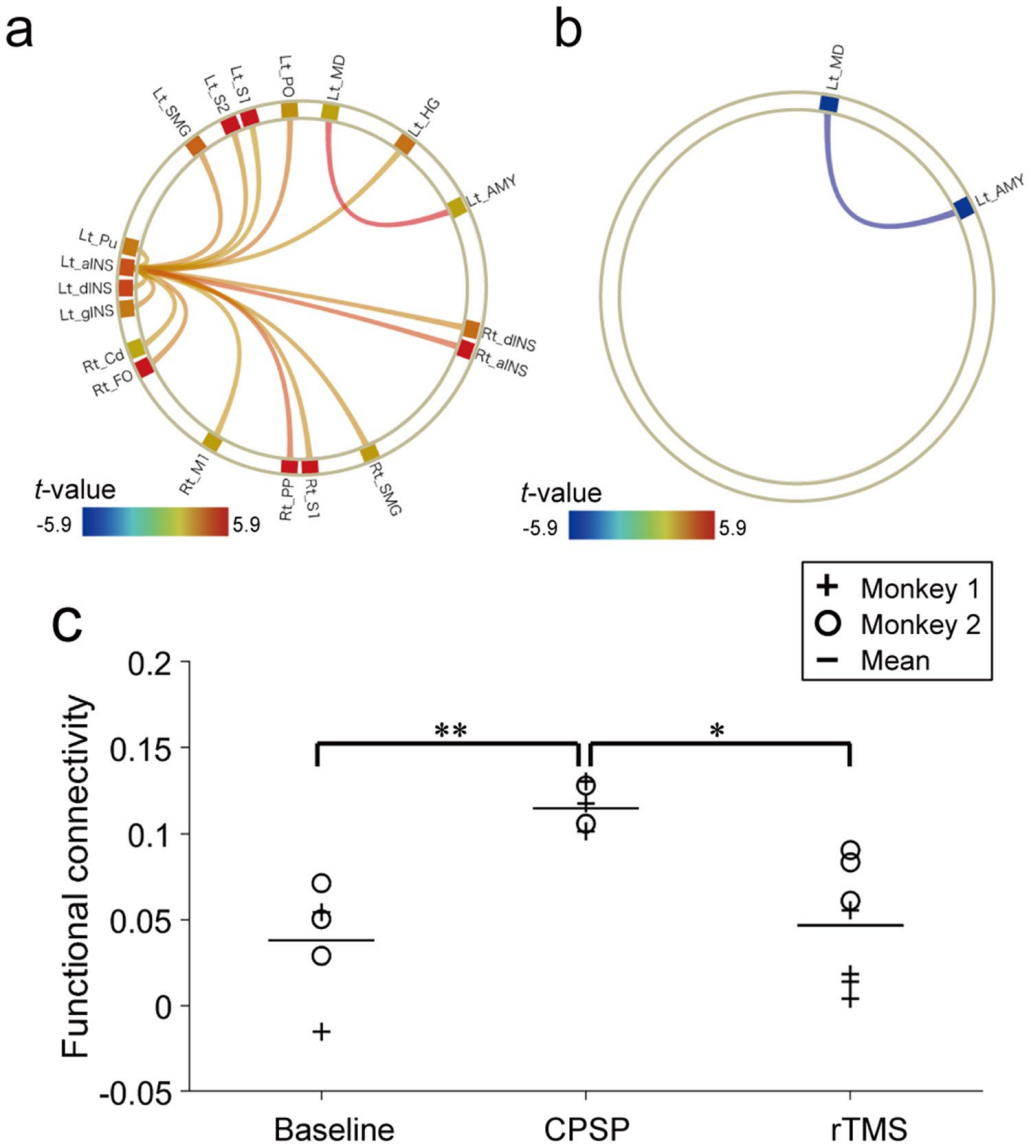
Changes in resting-state functional connectivity obtained from ROI-to-ROI analysis. (**a**) Changes in functional connectivity between the baseline and after CPSP development and (**b**) between the CPSP and after rTMS treatment. The red and blue lines indicate increased and decreased functional connectivity, respectively. The graphical figures are made by Conn toolbox. (**c**) The values of the functional connectivity between the left amygdala and the left MD for the two monkeys are shown. *p < 0.005, **p < 0.00005 after Bonferroni correction. *aINS* agranular insular cortex, *AMY* amygdala, *Cd* caudate, *CPSP* central poststroke pain, *dINS* dysgranular insular cortex, *FO* frontal operculum, *gINS* granular insular cortex, *HG* Heshcel’s gyrus, *Lt* left, *M1* motor cortex, *MD* mediodorsal nucleus of the thalamus, *PO* Parietal operculum cotex, *PP* planum polare, *Pu* putamen, *ROI* region of interest, *Rt* right, *rTMS* repetitive transcranial magnetic stimulation, *S1* primary somatosensory cortex, *S2* secondary somatosensory cortex, *SMG* supramarginal gyrus.

We investigated the changes in functional connectivity after CPSP development and rTMS administration by selecting several pain-related regions of interest (ROIs) and conducted ROI-to-ROI analysis. In the comparison between the baseline and after CPSP development, the functional connectivity tended to be increased among many pain-related ROIs (Fig. 4a and Table 1). One characteristic change was the increased functional connectivity between the agranular insula of the lesioned hemisphere (left) and various brain regions in both hemispheres. In addition, the most significant change was the connectivity between the mediodorsal nucleus (MD) of the thalamus of the lesioned hemisphere and the amygdala of the lesioned hemisphere (*t*_(16)_ = 5.92, uncorrected *p* = 0.00002, *p*-FDR = 0.001). Moreover, in the comparison between CPSP and rTMS conditions, decrease in the raised connectivity between the MD and amygdala was observed (Fig. 4b and Table 1; *t*_(16)_ = − 4.04, uncorrected *p* = 0.0009, *p*-FDR = 0.049). This trend occurred in both monkeys and the connectivity values between the MD and amygdala were significantly different between baseline and CPSP conditions and between CPSP and rTMS conditions (Fig. 4c). Although we administered rTMS on the left M1, no significant change in functional connectivity with the left M1 could be found. In the comparison between the baseline and rTMS conditions, ROI-to-ROI analysis showed no significant change in functional connectivity, and no significant difference in functional connectivity between the MD and amygdala.

**Table 1:** The statistical values in ROI-to-ROI analysis.

	ROI-1	ROI-2	*t*(16)	Uncorrected *p*-value	*p*-FDR
CPSP > Baseline	aINS (L)	dINS (L)	2.98	0.009	0.0406
		gINS (L)	3.16	0.006	0.0397
		S1 (L)	2.77	0.014	0.0455
		S2 (L)	3.07	0.007	0.0397
		SMG (L)	3.35	0.003	0.0397
		PO (L)	3.63	0.002	0.0395
		HG (L)	2.94	0.010	0.0406
		Pu (L)	2.77	0.014	0.0455
		aINS (R)	4.44	< 0.001	0.0121
		dINS (R)	3.13	0.006	0.0397
		S1 (R)	3.06	0.007	0.0397
		M1 (R)	2.77	0.014	0.0455
		SMG (R)	3.06	0.007	0.0397
		PP (R)	4.39	< 0.001	0.0121
		FO (R)	3.27	0.003	0.0397
		Cd (R)	2.92	0.010	0.0406
	MD (L)	AMY (L)	5.92	< 0.001	0.0010
rTMS > CPSP	MD (L)	AMY (L)	-4.04	< 0.001	0.0490

The *p*-values and *t*-values of the functional connectivity that significantly changed in the ROI-to-ROI analysis. The significance level was the ROI-level adjusted for multiplicity (*p*-FDR < 0.05).

*aINS* agranular insular cortex, *AMY* amygdala, *Cd* caudate, *CPSP* central poststroke pain, *dINS* dysgranular insular cortex, *FDR* false discovery rate, *FO* frontal operculum, *gINS* granular insular cortex, *HG* Heshcel’s gyrus, *L* left, *M1* motor cortex, *MD* mediodorsal nucleus in thalamus, *PO* Parietal operculum cortex, *PP* planum polare, *Pu* putamen, *ROI* region of interest, *R* right, *rTMS* repetitive transcranial magnetic stimulation, *S1* primary somatosensory cortex, *S2* secondary somatosensory cortex, *SMG* supramarginal gyrus.

**Figure 5:**
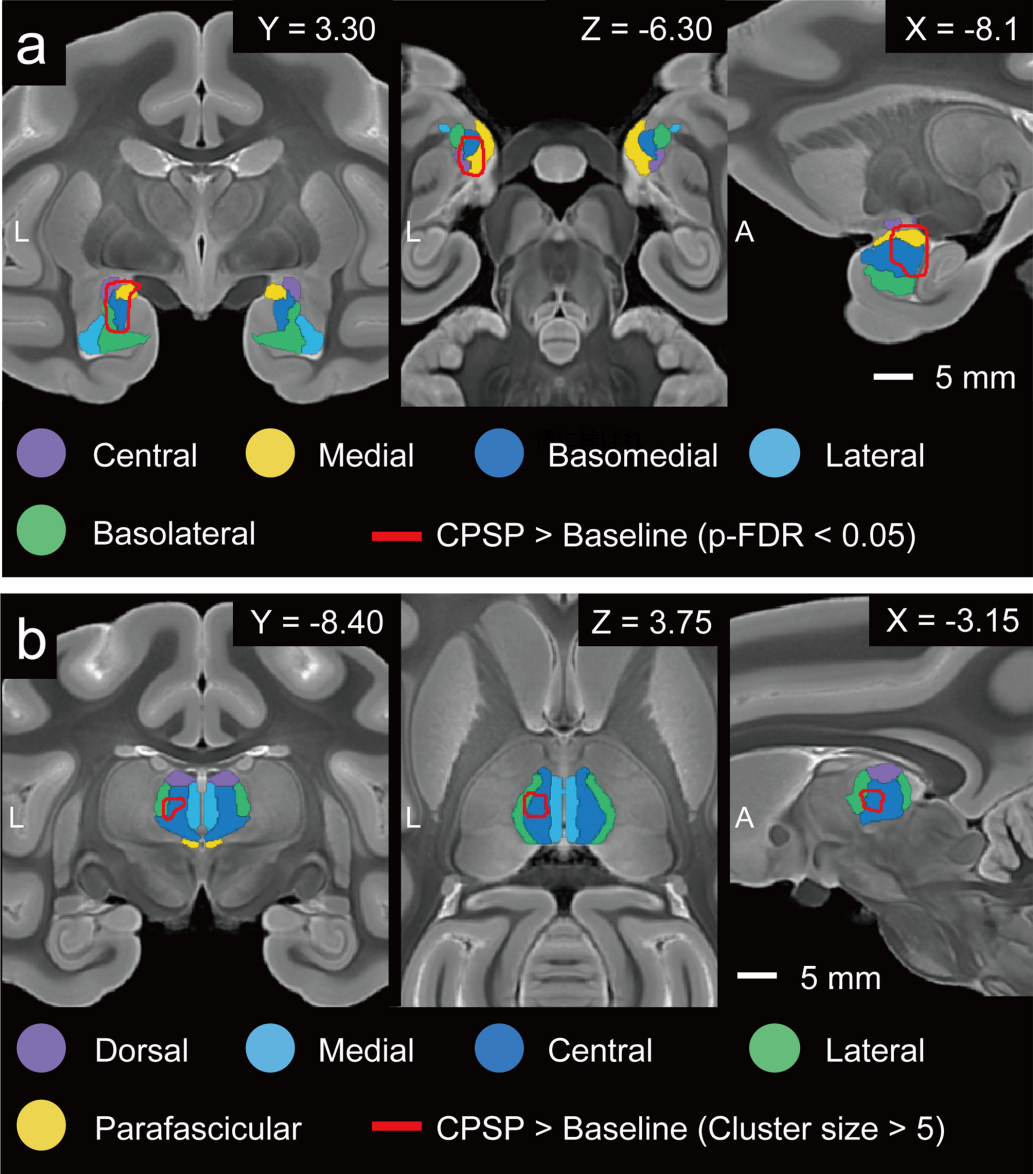
Detailed connectivity between the mediodorsal nucleus (MD) of the thalamus and amygdala obtained from seed-to-voxel analyses. Coronal (left), axial (centre), and sagittal (right) slices of the T2-weighted macaque atlas are shown. (**a**) Results of seed-to-voxel analysis when the MD is selected as the seed. The red lines indicate the edge of the voxel clusters in which the functional connectivity is significantly changed (voxel level uncorrected *p* < 0.001 and cluster level *p*-FDR < 0.05). The coloured areas indicate subregions of the amygdala drawn by using the template atlas. (**b**) Results of seed-to-voxel analysis when the amygdala is selected as the seed. We adjusted the significance level of the cluster size to detect focal changes (cluster size > 5). Coloured areas indicate the nucleus of the thalamus, drawn by using the template atlas. The xyz coordinate is defined at the anterior commissure. *A* anterior, *CPSP* central poststroke pain, *L* left.

Seed-to-voxel analysis was then conducted to investigate the functional connectivity between the MD and amygdala in detail. When the MD was selected as a seed (Fig. 5a), the voxels with higher connectivity after CPSP development were approximately overlapped with the basomedial nucleus of the amygdala (voxel-level *p*-uncorrected < 0.001 and cluster size *p*-FDR < 0.05). On the other hand, when the amygdala was selected as a seed, we found no significant changes in functional connectivity after CPSP development. We presumed that this result may be because the MD is very small and that changes in functional connectivity occurred focally. To detect focal changes, we adjusted the significance level of the cluster size (i.e., cluster size > 5). We found significant changes at the central part of the MD (Fig. 5b). We verified this result by conducting selected ROI-to-ROI analysis in which the central and lateral part of the MD and the basomedial and medial nucleus of the amygdala were the ROIs. The results of selected ROI-to-ROI analysis revealed a distinct difference between the baseline and CPSP conditions (*p* = 0.00001, *t*_(16)_ = 6.35, and the difference of functional connectivity between the baseline and CPSP increased from 0.0773 to 0.0904). In the comparison between the CPSP and after rTMS and between the baseline and after rTMS, no significant change existed in functional connectivity in seed-to-voxel analysis. Based on these results, we concluded that the connection between the basomedial and medial nucleus in the amygdala and the central and lateral part of the MD may be associated with CPSP development.

## Discussion

**Figure 6:**
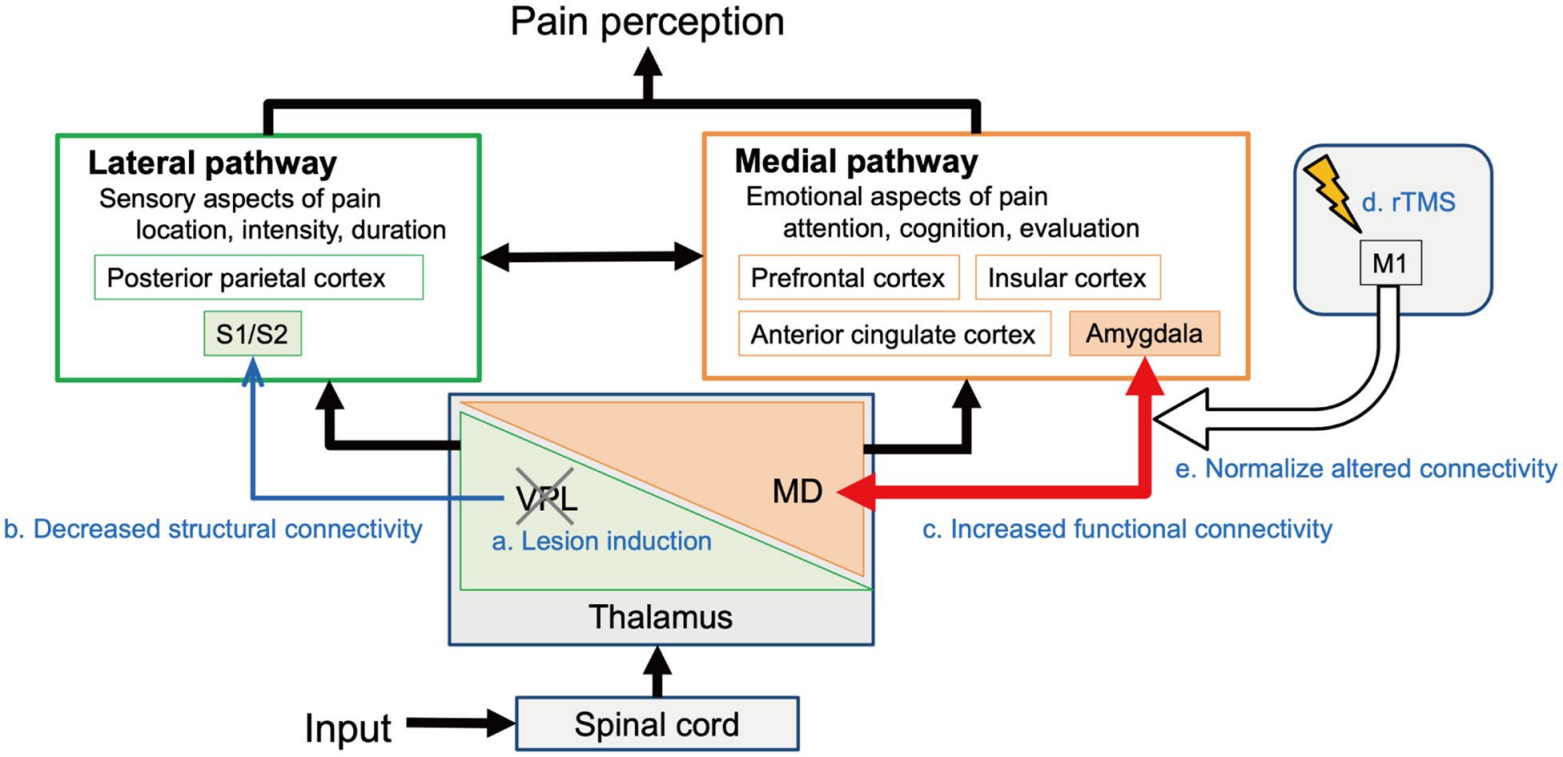
Structural and functional changes related to central poststroke pain (CPSP) development and rTMS treatment in the pain-related regions. Brain areas involved in pain processing can be divided into lateral and medial pathways. Monkeys developed CPSP after a lesion was induced in the VPL in the lateral pathway. After the CPSP development, the structural connectivity between the VPL and the S1/S2 decreased. However, changes in functional connectivity occurred in the medial pathway. The most significant change was an increase in the functional connectivity between the MD and amygdala. After the rTMS treatment, the altered functional connectivity between the MD and amygdala was normalized. These results suggest that altered functional connectivity between the MD and amygdala is one possible cause of the CPSP after a VPL lesion, and that rTMS therapy alleviates the abnormal connectivity and thereby may contribute to the therapeutic effect of rTMS. *M1* primary motor cortex, *MD* mediodorsal nucleus in the thalamus, *S1* primary somatosensory cortex, *S2* secondary somatosensory cortex, *rTMS* repetitive transcranial magnetic stimulation, *VPL* ventral posterolateral nucleus in the thalamus.

We investigated the anatomical and functional brain changes occurring from baseline to CPSP development and with rTMS administration by using CPSP model monkeys. The findings are summarized in Fig. 6. First, the monkeys showed late-onset hyperalgesia caused by lesions in the VPL; treatment with 5-Hz rTMS on the ipsilesional M1 suppressed it. Second, the structural connectivity in the white matter between the thalamus and the somatosensory cortex on the lesioned side decreased after the lesions. Third, after CPSP development, the functional connectivity increased between the ipsilesional MD and amygdala, and between the ipsilesional agranular insular and various pain-related regions. Finally, with rTMS, the increased connectivity between the MD and amygdala decreased to the baseline level. Based on these results, we concluded that the unusually strengthened functional connectivity between the MD and amygdala is a possible cause of CPSP, and that the analgesic effect of rTMS occurs by normalizing the altered functional connectivity.

CPSP occurs in 1–12% of stroke patients and has a negative impact on the quality of life. A stroke in the lateral somatosensory pathway can lead to late-onset CPSP. High-frequency rTMS on M1 is a promising treatment for CPSP patients^11,12^. However, the pathophysiology of CPSP and therapeutic mechanism of rTMS remain unclear. In this animal study, we developed CPSP model monkeys by lesions in the VPL, that consists of the lateral somatosensory pathway, which is the same method of the previous study^14^, and our two monkeys reproduced late-onset hyperalgesia and the changes in the pain threshold persisted at least five months (Fig. 2). This changes in pain perception of monkeys did not report after the lesion in the other brain areas. The late-onset characteristic faithfully resembles the clinical CPSP symptoms.

Furthermore, we demonstrated that the same rTMS parameter used for CPSP patients also had therapeutic effects on CPSP model monkeys (Fig. 2). Thus, we presume that the developmental mechanism of CPSP and therapeutic mechanism of rTMS in the CPSP model monkeys may reproduce the human CPSP. Based on these results, in addition to therapeutic research for CPSP, the CPSP model monkey is helpful for investigating the mechanism of CPSP development and the mechanism of the rTMS treatment in human CPSP.

For structural connectivity, studies in CPSP patients revealed decrease of fibres in the thalamocortical somatosensory pathway^16–20^. In this animal study, we also found decreased structural connectivity between the ipsilesional VPL and the primary somatosensory cortex (S1) and secondary somatosensory cortex (S2) (Fig. 3), that form the spinothalamic tract for sensory processing^21^. Particularly in pain perception, the projection from VPL to S1/S2 is the lateral pathway and conveys information about the location and intensity of painful stimulation (Fig. 6). Goto et al.^22^ reported that rTMS-effective CPSP patients had a higher delineation ratio of the fibres between the VPL and S1/M1 than did rTMS-ineffective CPSP patients. They reported that rTMS efficacy is influenced by the amount of remaining thalamocortical fibres after a stroke in the somatosensory pathway. In our study, the connectivity in the thalamocortical pathway was decreased but remained, even after the thalamic lesions (Supplementary Fig. S2); thus, rTMS could possibly have clinical effects through this pathway. In this study, we found no significant effect of the rTMS on the structural connectivity going through the thalamus. A previous study assessed white matter structural intensity during pain relief in a human tonic pain model by using another type of neuromodulation, transcranial direct current stimulation, on the dorsolateral prefrontal cortex^23^. The investigators reported possible structural changes after the stimulation. The difference between the present and previous results may reflect differences in species, stimulation methods, and location.

Scientists have hypothesized that the maladaptive reorganization of the pain network is responsible for CPSP, and that a neuromodulation technique could normalize it and have a therapeutic effect^5^. In the current study, we compared the resting-state functional connectivity to investigate maladaptive reorganization of the pain network. The ROI-to-ROI analysis revealed an increase in functional connectivity among the regions associated with pain after CPSP development (Fig. 4). Some increased connectivity was observed in the network associated with memory, emotion, and reward, classified as medial pathways in pain perception, although we created a lesion in the VPL regarding it as part of lateral pain pathway. The most significant increases in functional connectivity were between the MD and the amygdala, both included in the medial pathways in pain perception^24–26^. Previous human studies also report an increase in functional connectivity with the MD or the amygdala in clinical research between healthy individuals and individuals with pain other than CPSP such as complex regional pain syndrome and chronic headache^27,28^. In this study, we showed changes in pain threshold and changes in functional connectivity in the same individuals. Moreover, the rTMS to CPSP model monkeys suppressed hyperalgesia (Fig. 2) and normalized the altered functional connectivity between the MD and the amygdala (Fig. 4). Although it remains unclear how the effect of rTMS spreads from the stimulated M1 to the pain-related regions, we showed that the M1 rTMS could affect functional connectivity in deep brain regions. This finding seems consistent with the hypothesis that rTMS directly affects the dysfunctional reorganization of the pain network, which may cause CPSP^5^. These findings support the hypothesis that large-scale functional reorganization of the pain network underlies CPSP development and rTMS therapeutic mechanism.

Seed-to-voxel analysis further revealed focal changes in connectivity between the central part of the MD and basomedial nucleus of the amygdala (Fig. 5). However, in the seed-to-voxel analysis, we could not find the effect of the rTMS, that observed in the ROI-to-ROI analysis. This might reflect the rather spreading effects of rTMS on the functional brain connectivity. Basomedial nucleus of the amygdala, also known as the accessory basal nucleus, is a part of basolateral amygdala groups and contains neurons that respond to painful stimuli^29^. Basomedial nucleus of the amygdala is bi-directionally connected with the prefrontal cortex and projects mainly to the medial, not the central part of the MD^30^. Contributions of the central part of the MD, also known as the parvocellular subdivision of the MD, to higher cognitive functions were reported^31^. Future study will therefore need to consider the specific functional interaction of the central part of the MD and basomedial nucleus of the amygdala in pain processing.

A previous study using the same CPSP model monkeys reported that painful stimulation induced changes in the blood oxygenation level-dependent response in pain-related brain areas, including the posterior insular cortex, S2, anterior cingulate cortex (ACC), and amygdala^32^. Similar results were also reported in studies comparing pain response in healthy individuals and individuals with chronic pain^33^. The MD and amygdala have connectivity with the frontal cortex including ACC^34–37^, which is associated with the assessment and expression of negative emotion^38^. The working hypothesis is that the unusually strong functional connectivity between the MD and the amygdala causes an incorrect appraisal of painless sensory input in medial pathway including ACC and produces the CPSP condition.

Our study had several limitations. We used two CPSP model monkeys, but more subjects are necessary for reproducible results, and control subjects with brain lesion but no CPSP are important for understanding human CPSP development after the stroke. Some differences existed in the artificial lesions of the haemorrhages and how the hyperalgesia manifested. These individual differences possibly affected the results. The pain perception measured in this study was pain induced by stimulations, but this pain is not strictly the same as the persistent pain experienced by CPSP patients. We did not use the pain rating scale commonly used for human CPSP because such subjective measures could not be obtained from the monkeys. Also, our behavioural measures could not distinguish the changes in pain perception and sensory perception, and thus could not precisely evaluate the sensory dysfunction that is usually observed in human CPSP development. The rTMS parameters and its site were the same as for CPSP patients, but more studies are needed to see the analgesic effects in different parameters. In this study, we did not focus on the duration of rTMS effects and the changes in the brain function or in the behavioural tasks induced by rTMS compared to healthy control. To reduce the noises caused by head movements, we used anaesthetics during the MRI scan. However, these drugs influence the activity of brain regions associated with pain. In this study, we used propofol as the anaesthetic, as in a recent fMRI study using primate CPSP models^32^. Propofol is considered to have few analgesic effects^39^; however, we cannot ignore the effects of anaesthetics on the brain function. For defining the brain regions, we used the atlas for the *Macaca mulatta*^15^, but we did not correct for differences in the brains of *Macaca fuscata*. Moreover, the regions of interest were selected to include the regions which were assumed to be associated with pain perception in humans. Whether these regions in monkeys are the same as humans is unclear. Another issue is whether the reduction in functional connectivity between the MD and amygdala with rTMS is specific effect to CPSP model monkeys or similarly affects intact monkeys. A previous systematic review assessing the effects of rTMS on functional connectivity in clinical and healthy control populations highlighted that some individuals had an increase whereas others had a decrease in functional connectivity after rTMS; this finding possibly depends on the nature of the clinical population^40^. These challenges are for future research.

In conclusion, based on the findings of this animal study, we found an increase in functional connectivity between the MD of the thalamus and amygdala in the CPSP condition, compared to the control condition. Applying 5-Hz rTMS on the ipsilesional primary motor cortex raised the pain threshold of CPSP monkeys. In the condition with rTMS, the strength of the MD–amygdala connectivity dropped to the control level. These results suggested that altered connectivity between the MD and amygdala is a possible cause of the CPSP after a VPL lesion, and that rTMS therapy alleviated the abnormal connectivity. This factor may contribute to the therapeutic effect of rTMS.

## Materials and methods

### Study overview

We quantified the pain threshold of monkeys by a behavioural experiment and determined functional and anatomical changes in the brain using MRI analysis in the baseline condition, after CPSP development, and with rTMS. After collecting baseline data, we induced an artificial stroke in the unilateral VPL to create CPSP model monkeys. We then applied rTMS to monkeys by using the same parameters as for human CPSP patients.

All experimental procedures were approved by the Committee for Animal Experiments at Osaka University and National Bio-Resource Project (NBRP), and were carried out in accordance with the National Institutes of Health Guidelines for the Care and Use of Laboratory Animals.

### CPSP model monkeys

We used two adult male Japanese monkeys (*Macaca fuscata*; weight at surgery: 8.0 kg for monkey 1 and 7.4 kg for monkey 2) provided by the NBRP-Nihonzaru at Kyoto University Primate Research Institute (Inuyama, Japan) with support in part by NBRP of the Japan Agency for Medical Research and Development (Tokyo, Japan).

We induced a local lesion within the VPL by collagenase injection, as previously described^14^. The collagenase was injected into the left VPL of both monkeys. We used the contralateral side to the hand with a higher pain threshold to examine the changes in pain threshold more accurately. Details of the lesion procedures are described in Supplementary Method.

### rTMS protocol

We used MagStim Super Rapid^2^ Plus^1^ (Magstim Company Ltd., Whitland, UK) stimulation system and D70 Alpha Flat Coil (Magstim Company Ltd.) of figure-eight coil. The rTMS was applied at 90% motor threshold that stimulation moved the monkeys’ fingers with an approximately one-half possibility. Stimuli were delivered at the pulse frequency of 5 Hz, each pulse train of 10 s, and intertrain interval of 50 s. Trains were repeated 10 times, and finally generated 500 pulse stimulations (see also Supplementary Method). This stimulation parameter is equivalent to that used for patients with CPSP^41–45^. Sham stimulation was performed by tilting the same coil 90 degrees and the coil was also separated (by more than 20 mm) from their heads.

### Behavioural tasks

The withdrawal thresholds of the mechanical and thermal stimulation were measured to evaluate the pain perception. The behavioural tasks were conducted, as previously described^14^ (see also Supplementary Method). Mechanical threshold was measured by using electric von Frey Anesthesiometer (IITC Life Science, Inc., Woodland Hills, CA, USA) which included rigid, #10, #13 and #15 filaments. The withdrawal pressure for the second, third and fourth fingers of both hands were recorded. Thermal threshold was recorded using thermal stimulator (SCP-85; AS ONE Corporation, Osaka, Japan) in which the surface was maintained at 37 °C as the control, 45–50 °C as the hot stimuli, and 5–15 °C as the cold stimuli (the pre-lesion data for monkey 2 at 5 °C was lacking). The withdrawal latency of both palms at these temperatures was respectively measured. After the monkeys showed CPSP in these results, the behavioural tasks were conducted just after application of real or sham rTMS.

The behavioural data were analysed by using nonparametric tests such as the Kruskal–Wallis test with Dunn’s post hoc analysis and Mann–Whitney *U* test (Fig. 2a,c), and the Wilcoxon signed-rank test (Fig. 2b,d; two-sided *p* < 0.05 was significant). The analysis was conducted using software JMP pro 14, (SAS Institute Inc., Cary, NC, USA).

### MRI data acquisition

Structural MRI, DTI, and resting-state functional MRI (rs-fMRI) scans were conducted using a 7-T MR scanner (7 T Magnetom; Siemens Healthineers, Erlangen, Germany) with a 32-channel head coil (Nova Medical, Wilmington, MA, USA). The monkeys were anesthetized by a continuous intravenous injection of propofol (0.3–0.4 mg/kg/min) through an indwelling needle inserted in their leg. The heart rate was monitored and maintained within 70–110 bpm (60–80% of the wake rate). The heads were fixed by using headrest composed of urethane foam that was individually moulded to their heads. The airway was maintained by an intubation tube. MRI data were obtained before CPSP development as baseline condition, and after CPSP development with sham stimulation and rTMS treatment (monkey 1: 14–23 weeks; monkey 2: 15–23 weeks after the lesion). The rTMS or sham stimulation was administered before sedation in the same manner for the behavioural experiment. The MRI scan was started approximately 1 h after completing the stimulation. To eliminate the cumulative rTMS effects over time, we did not apply rTMS for at least 2 days prior to MRI, and days that MRI scanning with rTMS occurred were not consecutive. The analysed data of monkey 1 were two images for the baseline, three images for CPSP, four images for rTMS, and the analysed data of monkey 2 were three images for each condition.

For the anatomical images (field of view [FOV], 128 × 128 mm; matrix, 192 × 192; slice thickness, 0.7 mm; number of slices, 128; voxel size, 0.67 × 0.67 × 0.70 mm), we obtained T1-weighted images (repetition time/echo time [TR/TE], 2200/1.94 ms; flip angle [FA], 5°) and T2-weighted images (TR/TE, 3200/409 ms; FA, 120°). The pulse sequence of rs-fMRI was TR/TE, 1000/20 ms; FA, 55°; FOV, 150 × 150 mm; matrix, 100 × 100; slice thickness, 1.5 mm; number of slices 42; voxel size, 1.5 × 1.5 × 1.5 mm; and acquired for 30 min (one data set of the baseline data for monkey 1 was for 20 min). The pulse sequence of DTI was TR/TE = 5000/61 ms; FA, 90°; FOV = 150 × 150 mm; matrix, 96 × 96; number of slices, 44; slice thickness = 1.6 mm; voxel size, 1.562 × 1.562 × 1.6 mm. In DTI, we used the gradients of 30 directions at the conditions of b = 1000 s/mm^2^ and b = 2000 s/mm^2^. We also took two b = 0 images with opposite phase encoding directions for distortion correction.

### MRI analysis

To determine the haemorrhagic lesion site, we used T1-weighted images. We used the DTI-based atlas of rhesus monkey (*Macaca mulatta*)^15^ (http://www.civm.duhs.duke.edu/rhesusatlas/). The transformation was calculated by using advanced normalization toolboxes (ANTs, http://stnava.github.io/ANTs/). We first applied the VPL image from the atlas to the subject images (Fig. 1a,b). Then, we compared the lesion area of both monkeys in the atlas space. (Fig. 1c).

To analyse anatomical changes in the brain, tractography analysis was performed using FMRIB Diffusion Toolbox (FDT) in FMRIB Software Library (FSL v5.0.1; University of Oxford, Oxford, England) (Fig. 3). The numbers of reconstructed streamlines that passed through the entire thalamus were tracked and analysed as the index of structural connectivity. The significance level was set at *p* < 0.001 (uncorrected) for the voxel level and *p*-FWE < 0.05 for the cluster size. Details of the DTI procedures are described in Supplementary Method.

ROI-to-ROI analysis was conducted to determine the changes in functional connectivity among ROIs that are associated with chronic pain^33,46,47^ (Fig. 4, Table 1). We used the 54 ROIs from the rhesus macaque atlas^15^ (details are shown in Supplementary Table S1). Based on the human regions associated with chronic pain, we selected macaque ROIs from neurosynth.org (https://www.neurosynth.org). Moreover, two ROIs associated with the default mode network were included. If an ROI in the human atlas was split in the macaque atlas, we combined some regions and used one ROI. For example, the amygdala is split into 14 nuclei in the atlas; therefore, we considered them as a single ROI ‘amygdala’.

Before the analysis, rs-fMRIs were preprocessed for motion correction and normalization with ANTs and smoothed by SPM12. To transform the fMRIs and to correct the motion of the heads, we created a time-averaged image. Motion correction parameters were as follows: metric, Mutual Information; transform, Rigid; and gradient step, 0.1. The rs-fMRI images were registered to time-averaged images. We then calculated the transformations from the time-averaged images to the subject T2-weighted images and those from the subject T2-weighted images to the atlas b0 image (radius, 4; spine distance, 26) in ANTs. The normalized images were obtained from the images applied to the transforms and were smoothed by using the Gaussian kernel in SPM12. Full width at half maximum of smoothing was 3 mm. We ran ROI-to-ROI analysis in MATLAB (MathWorks, Natick, MA, USA) with the CONN toolbox ver.17c (Gabrieli Lab at MIT McGovern Institute for Brain Research, Cambridge, MA; https://web.conn-toolbox.org). To remove the individual differences and changes over time in functional connectivity, we included the subject-varying and time-varying covariates. For denoising, we selected ‘Effect of rest’ and ‘cerebrospinal fluid’ in the confounds. Finally, the band-pass filter (0.01–0.18 Hz) was temporally applied in the functional MR images.

In the statistical analysis, we conducted an analysis of covariance (ANCOVA). We compared the changes in functional connectivity between the baseline and CPSP, and between CPSP and rTMS conditions. The significance level was ROI-level adjusted for multiplicity (*p*-FDR < 0.05). We also ran seed-to-voxel analysis to determine detailed connectivity within the ROIs (Fig. 5). The datasets and covariates for seed-to-voxel analysis were the same as in the ROI-to-ROI analysis.

## Supplementary Information

Supplementary Information.

## Data Availability

The datasets generated during the current study are available from the corresponding author on reasonable request.

## References

[1] Sprenger T (2012). Assessing the risk of central post-stroke pain of thalamic origin by lesion mapping. Brain.

[2] Klit H, Finnerup NB, Jensen TS (2009). Central post-stroke pain: Clinical characteristics, pathophysiology, and management. Lancet Neurol..

[3] Kumar B, Kalita J, Kumar G, Misra UK (2009). Central poststroke pain: A review of pathophysiology and treatment. Anesth. Analg..

[4] Vartiainen N (2016). Thalamic pain: Anatomical and physiological indices of prediction. Brain.

[5] Hosomi K, Seymour B, Saitoh Y (2015). Modulating the pain network—Neurostimulation for central poststroke pain. Nat. Rev. Neurol..

[6] Fagundes-Pereyra WJ (2010). Motor cortex electric stimulation for the treatment of neuropathic pain. Arq. Neuropsiquiatr..

[7] Tanei T (2011). Efficacy of motor cortex stimulation for intractable central neuropathic pain: Comparison of stimulation parameters between post-stroke pain and other central pain. Neurol. Med. Chir. (Tokyo).

[8] Nandi D (2002). Peri-ventricular grey stimulation versus motor cortex stimulation for post stroke neuropathic pain. J. Clin. Neurosci..

[9] Hosomi K (2008). Electrical stimulation of primary motor cortex within the central sulcus for intractable neuropathic pain. Clin. Neurophysiol..

[10] Lazorthes Y, Sol JC, Fowo S, Roux FE, Verdié JC (2007). Motor cortex stimulation for neuropathic pain. Acta Neurochir. Suppl..

[11] Lefaucheur J-P (2014). Evidence-based guidelines on the therapeutic use of repetitive transcranial magnetic stimulation (rTMS). Clin. Neurophysiol..

[12] O’Connell NE, Wand BM, Marston L, Spencer S, Desouza LH (2010). Non-invasive brain stimulation techniques for chronic pain. Cochrane Database Syst. Rev..

[13] Boes AD (2015). Network localization of neurological symptoms from focal brain lesions. Brain.

[14] Nagasaka K, Takashima I, Matsuda K, Higo N (2017). Late-onset hypersensitivity after a lesion in the ventral posterolateral nucleus of the thalamus: A macaque model of central post-stroke pain. Sci. Rep..

[15] Calabrese E (2015). A diffusion tensor MRI atlas of the postmortem rhesus macaque brain. Neuroimage.

[16] Jang SH, Lee J, Yeo SS (2017). Central post-stroke pain due to injury of the spinothalamic tract in patients with cerebral infarction: A diffusion tensor tractography imaging study. Neural Regen. Res..

[17] Jang SH, Kim J, Lee HD (2018). Delayed-onset central poststroke pain due to degeneration of the spinothalamic tract following thalamic hemorrhage: A case report. Medicine (Baltimore).

[18] Hong JH (2010). Injury of the spino-thalamo-cortical pathway is necessary for central post-stroke pain. Eur. Neurol..

[19] Hong JH (2012). The prevalence of central poststroke pain according to the integrity of the spino-thalamo-cortical pathway. Eur. Neurol..

[20] Li, X., Feng, Y. & Gao, F. Maladaptive reorganization in pain-related brain network contributing to the central post-stroke pain. **9**, 2186–2197 (2019).

[21] Willis WD, Zhang X, Honda CN, Giesler GJ (2002). A critical review of the role of the proposed VMpo nucleus in pain. J. Pain.

[22] Goto T (2008). Diffusion tensor fiber tracking in patients with central post-stroke pain; Correlation with efficacy of repetitive transcranial magnetic stimulation. Pain.

[23] Lin RL (2017). Structural connectivity variances underlie functional and behavioral changes during pain relief induced by neuromodulation. Sci. Rep..

[24] Aggleton JP, Mishkin M (1984). Projections of the amygdala to the thalamus in the cynomolgus monkey. J. Comp. Neurol..

[25] Miyashita T, Ichinohe N, Rockland KS (2007). Differential modes of termination of amygdalothalamic and amygdalocortical projections in the monkey. J. Comp. Neurol..

[26] Mitchell AS, Chakraborty S (2013). What does the mediodorsal thalamus do?. Front. Syst. Neurosci..

[27] Schwedt TJ (2013). Atypical resting-state functional connectivity of affective pain regions in chronic migraine. Headache.

[28] Simons LE (2014). The responsive amygdala: Treatment-induced alterations in functional connectivity in pediatric complex regional pain syndrome. Pain.

[29] Ji G (2010). Cognitive impairment in pain through amygdala-driven prefrontal cortical deactivation. J. Neurosci..

[30] Kelly, R. & Stefanacci, L. Amygdala: structure and circuitry in primates. In *Encyclopedia of Neuroscience* (ed. Squire, L. R.) 341–345 (Academic Press, 2009). 10.1016/B978-008045046-9.00148-0.

[31] Pergola G (2018). The regulatory role of the human mediodorsal thalamus. Trends Cogn. Sci..

[32] Nagasaka K, Takashima I, Matsuda K, Higo N (2020). Brain activity changes in a monkey model of central post-stroke pain. Exp. Neurol..

[33] Peyron R, Laurent B, García-Larrea L (2000). Functional imaging of brain responses to pain. A review and meta-analysis (2000). Neurophysiol. Clin..

[34] Aggleton JP, Wright NF, Rosene DL, Saunders RC (2015). Complementary patterns of direct amygdala and hippocampal projections to the macaque prefrontal cortex. Cereb. Cortex.

[35] Meda KS (2019). Microcircuit mechanisms through which mediodorsal thalamic input to anterior cingulate cortex exacerbates pain-related aversion. Neuron.

[36] Neugebauer V (2015). Amygdala pain mechanisms. Handb. Exp. Pharmacol..

[37] Reppucci CJ, Petrovich GD (2016). Organization of connections between the amygdala, medial prefrontal cortex, and lateral hypothalamus: A single and double retrograde tracing study in rats. Brain Struct. Funct..

[38] Etkin A, Egner T, Kalisch R (2011). Emotional processing in anterior cingulate and medial prefrontal cortex. Trends Cogn. Sci..

[39] Steinbacher DM (2001). Propofol: A sedative-hypnotic anesthetic agent for use in ambulatory procedures. Anesth. Prog..

[40] Beynel L, Powers JP, Appelbaum LG (2020). Effects of repetitive transcranial magnetic stimulation on resting-state connectivity: A systematic review. Neuroimage.

[41] Hirayama A (2006). Reduction of intractable deafferentation pain by navigation-guided repetitive transcranial magnetic stimulation of the primary motor cortex. Pain.

[42] Saitoh Y (2007). Reduction of intractable deafferentation pain due to spinal cord or peripheral lesion by high-frequency repetitive transcranial magnetic stimulation of the primary motor cortex. J. Neurosurg..

[43] Hosomi K (2013). Daily repetitive transcranial magnetic stimulation of primary motor cortex for neuropathic pain: A randomized, multicenter, double-blind, crossover, sham-controlled trial. Pain.

[44] Hosomi K (2013). Cortical excitability changes after high-frequency repetitive transcranial magnetic stimulation for central poststroke pain. Pain.

[45] Hosomi K (2020). A randomized controlled trial of 5 daily sessions and continuous trial of 4 weekly sessions of repetitive transcranial magnetic stimulation for neuropathic pain. Pain.

[46] Ingvar M (1999). Pain and functional imaging. Philos. Trans. R. Soc. Lond. B Biol. Sci..

[47] Apkarian AV, Bushnell MC, Treede R-D, Zubieta J-K (2005). Human brain mechanisms of pain perception and regulation in health and disease. Eur. J. Pain.

