# Supplementary material for: Repetitive transcranial magnetic stimulation restores altered functional connectivity of central poststroke pain model monkeys
Source: Sci Rep. 2021 Mar 17;11:6126. doi: 10.1038/s41598-021-85409-w (PMC7969937; doi:10.1038/s41598-021-85409-w)
Supplement: Supplementary file 1 — Supplementary Information. [file 41598_2021_85409_MOESM1_ESM.pdf]

Supplementary Information

Repetitive transcranial magnetic stimulation restores altered functional connectivity of central poststroke pain model monkeys

Yoshinori Kadono, Keigo Koguchi, Ken-ichi Okada, Koichi Hosomi, Motoki Hiraishi, Ikuhiro Kida, Takashi Ueguchi, Adnan Shah, Guoxiang Liu, Youichi Saitoh

Supplementary Method

Developing CPSP model monkeys

We used two adult male Japanese monkeys (*Macaca fuscata*). Monkeys were housed in individual primate box cages in the same room under 12-hour light/dark cycle, and humidity and temperature were monitored. Animals were fed standard monkey chow once a day and had free access to water. In addition, they were daily supplemented with fresh fruits or vegetables with positive interaction (e.g. hand feeding of treats) by the research staff.

We made two CPSP model monkeys by inducing a local lesion within the VPL by collagenase injection, based on the protocol of a previous report [1]. In detail, the injection site was determined by the T1-weighted MRI and by the microrecording of neuronal activity. Detailed MRI parameters are shown in the 'MRI data acquisition' section of the main text. For recording neuronal activity and injecting collagenase, the scalp above the left thalamus was removed and the recording chamber (made by Delrin) was attached on the head (10 mm anterior and 10 mm lateral from the centre of interaural line) using ceramic screw in aseptic surgery with general anaesthesia using a combination of medetomidine hydrochloride (0.1 mg/kg, i.m.) and ketamine hydrochloride (10 mg/kg, i.m.), and isoflurane (1~3%). During surgery, isoflurane levels, CO_2 levels, respiratory rate, electrocardiogram, pulse oximetry, blood pressure, and rectal temperature were monitored. The approximate position of the VPL was determined by the anatomical image. To identify the hand digits region of the VPL, we recorded neural activity by using tungsten microelectrode (impedance of 1–6 MΩ, FHC; Bowdoin, ME, USA) under light sedation. During the recording, the heart rate was maintained within 90–130 bpm (70%–90% of the wake rate) by controlling the dosage of propofol (0.1–0.2 mg/kg/min). We stimulated their right-hand fingers by using the hand-blush at each depth of the electrode and determined hand digits region of the left VPL. Collagenase type IV (C5138; Sigma, St. Louis, MO, USA; 200 U/mL in saline) was injected through the microsyringe into the two locations where 0.5 mm from the centre of the hand digits region in the VPL in the dorsoventral direction. At each injection site, 4 µL collagenase was injected for 4 minutes, and we waited for 10 minutes until moved to the next injection site.

Procedures and statistics for behavioural experiment

The behavioural tasks were performed, based on the protocol of a recent study [1]. During these tasks, the monkeys sat on a primate chair made of acrylate glass and their low backs and heads were fixed firmly. Their wrists were fixed to maintain their palm downside but not to prevent escaping backward from the stimulation. An eye shield was set on the chair and monkeys could not see their hands and stimulators during experiment. The mechanical threshold was measured by using electric von Frey Anesthesiometer (IITC Life Science, Inc., Woodland Hills, CA, USA), which included rigid, #10, #13 and #15 filaments. The data were excluded if the monkey intentionally pressed the filament or if its hand moved before filaments touched it sufficiently. The monkeys could get a food reward after the recording of each finger. The thermal threshold (45℃–50℃ as the hot stimulus and 5℃–15℃ as the cold stimulus) was recorded using thermal stimulator (SCP-85; As One Corporation, Osaka, Japan). To avoid burns, each trial took less than 1 minute. The recording from single temperature was repeated less than five trials and less than 90 seconds. Monkeys could get a food reward if they could endure the stimulus for 1 minute.

Both the mechanical and thermal tasks were conducted on the same day and twice weekly. The tasks were applied to one side at each condition, and then to the other side. The starting hand side was randomized to consider the concentration and the hunger of monkeys. The behavioural tasks were monitored by a video camera so that we could check the monkeys when they moved strangely or when we could not determine the withdrawal timing. After the monkeys showed CPSP, the behavioural tasks were conducted after an rTMS or a sham stimulation. Stimulation for the first day of a week was randomly assigned to rTMS or sham stimulation and the other was assigned for the rest day.

During rTMS treatment, monkeys sat on a monkey chair and the monkeys' heads were fixed by thermoplastic masks moulded to their heads. We determined stimulation site and intensity according to the humane research [2]–[4]. We performed single-pulse TMS on M1 of the lesion side and searched the hand area to which stimulus elicited the movement of the monkeys' fingers. The coil was firmly fixed by a manipulator to stimulate the hotspot. We determined 90% motor threshold stimulation that moved the monkeys' fingers with an approximately one-half possibility.

The pain threshold was analysed by collecting the data from multiple weeks to match conditions such as the starting side, the interval between rest days, and rTMS therapy. The sets of 4 weeks were combined, and the data sets were compared before creating the haemorrhage and every 4 weeks after creation. The first week after the lesion was excluded due to their recovery from surgery and medications (prestroke, 2–5 weeks, 6–9 weeks, 10–13 weeks, 14–17 weeks). After the onset of pain and the start of rTMS treatment, the data of sham stimulation was collected after a >2-day interval from a stimulation, to rule out the persistence of the effect of the rTMS therapy. For the mechanical threshold, the maximum value of each day for each finger was set as the withdrawal threshold value so that variation due to the number of trials and the order did not occur. The withdrawal threshold of each finger was compared with that of the opposite-side finger to calculate relative withdrawal threshold. For example, the maximum threshold value of the affected-side

second finger on one day divided by the maximum threshold value of the healthy-side second finger on that day. We collected the relative withdrawal threshold of the second, third, and fourth fingers, and treated these data with no distinction. The effect of rTMS therapy was verified by comparing the data with rTMS to the data without rTMS after pain onset. For the thermal threshold, the mean durable time until the escape at that temperature of one day was similarly used as the withdrawal threshold of affected hand. The relative withdrawal threshold was calculated as the withdrawal threshold of affected hand divided by the average of the endurance time on the other side of the 4-week period. That is, the ratios obtained by dividing the mean of the endurance time on the affected side at 50°C on one day by the average value of the endurance times at 50°C on the unaffected side of that period were used as the data set for that period. The sets of 4 weeks were compared in order and the effects of rTMS treatment were also verified after pain onset.

Procedures for DTI analysis

To analyse anatomical connectivity in the brain, tractography analysis was performed using FMRIB Diffusion Toolbox (FDT) in FMRIB Software Library (FSL v5.0.1, University of Oxford, Oxford, England; www.fmrib.ox.ac.uk/fsl) (**Fig. 3**). The streamlines that passed through the entire thalamus were tracked. The eddy current-induced distortions and subject movements were corrected using EDDY. Fractional-anisotropy (FA) images were obtained by running the DTIFIT. We then ran the Bayesian-estimation (bedpostx) (Fibres, 2; weight, 1; Burn in 1000). The seeds for tractography were determined by warping the thalamus images of both hemispheres, and the transformations from atlas b0 image to subject b0 images were calculated by ANTs. By using probabilistic tracking with crossing fibres (probtrackx), we made density maps of the streamlines that passed through the thalamus of the affected and unaffected sides. The parameters were (curvature threshold, 0.2; number of samples, 5000; number of steps per sample, 2000; steplength, 0.5 mm). To compare the density map in the template space, we warped the density maps by applying the transformations from subject FA images to the atlas FA image. The warped images were statistically analysed with one-way ANOVA by using SPM12 (The Wellcome Centre for Human Neuroimaging, London, UK) in MATLAB (MathWorks, Natick, MA, USA). The significance level was set as uncorrected $p < 0.001$ for voxel level and p-FWEc < 0.05 for cluster size.

Supplementary Figure S1

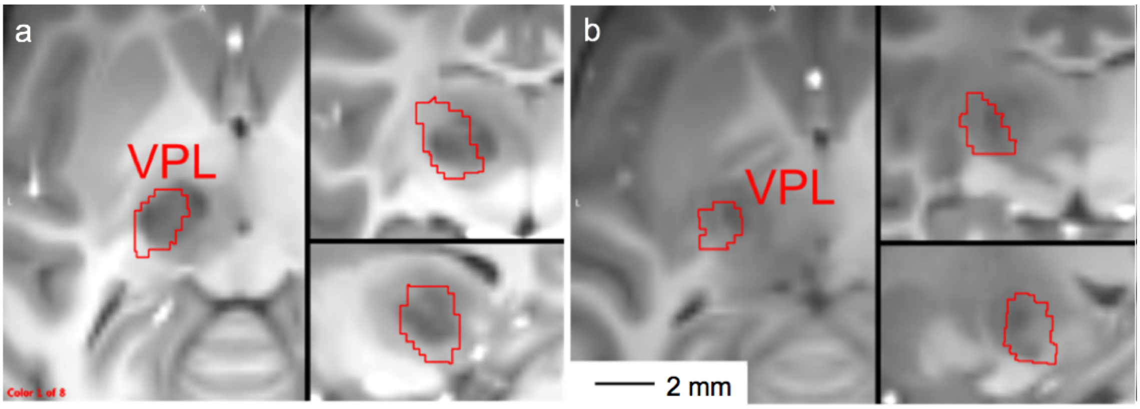

Induced lesion area on structural magnetic resonance images shortly after the injection. The T1-weighted magnetic resonance images of monkey 1 at 2 days (**a**) and monkey 2 at 3 days (**b**) after collagenase injection. The axial (left), coronal (upper right) and sagittal (lower right) slice images are shown. The red lines indicate the edge of the VPL for the lesion side warped from a monkey atlas. Vague lesions appear as low-intensity areas expanded for the whole VPL areas, accompanied by haematoma and oedema for surrounding structures involving the internal capsules adjacent to the VPL.

VPL: ventral posterolateral nucleus of the thalamus

Supplementary Figure S2

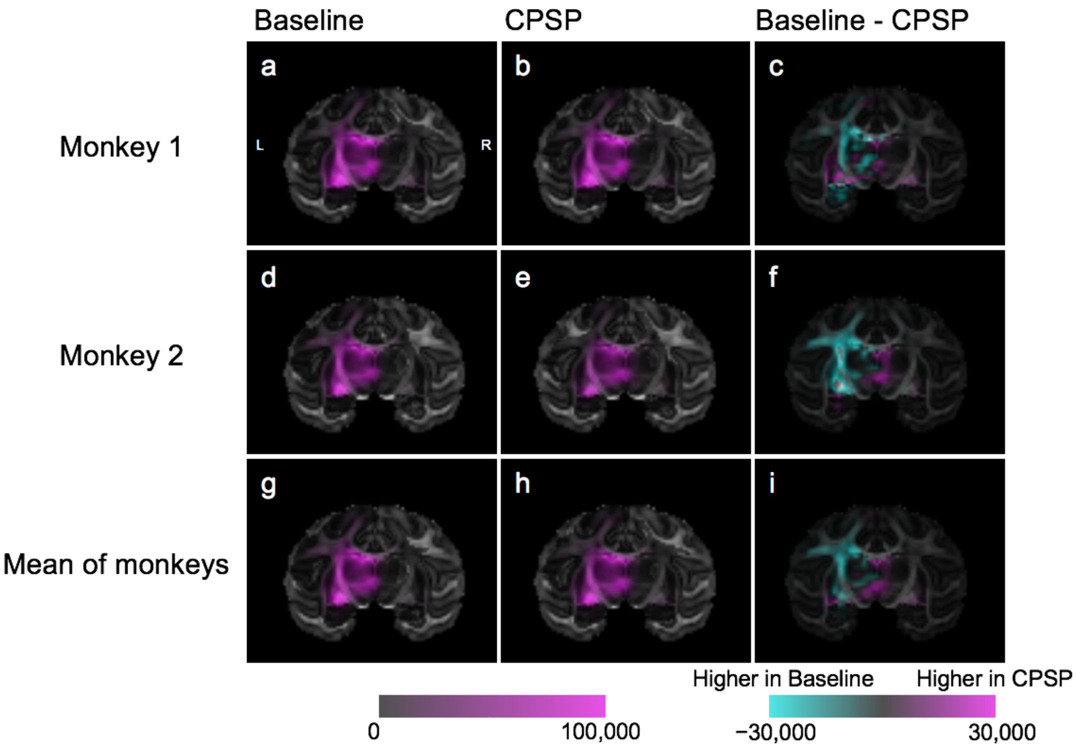

Comparison of the structural connectivity through lesion-side thalamus before and after CPSP development. The coronal slices obtained from the tractography analysis are shown. The left, middle, and right panels show the structural connectivity of baseline condition (**a, d,** and **g**), the CPSP condition (**b, e,** and **h**), and the difference between the baseline and CPSP conditions (**c, f,** and **i**), respectively. The structural connectivity between the lesion-side ventral posterolateral nucleus of the thalamus and the primary and secondary somatosensory cortex are decreased in both monkeys, but the streamlines are not completely lost.

CPSP: central poststroke pain

Supplementary Table S1

Classification		Abbreviation	Full name	Volume (mm^3)
Pain matrix	Lateral pathway	VP (L, R)	Thalamus, ventral posterolateral + posteromedial	297
		S1 (L, R)	Primary Somatosensory Cortex	2761
		S2 (L, R)	Secondary Somatosensory Cortex	1296
		gINS (L, R)	Granular insular cortex	557
	Medial pathway	MD (L, R)	Thalamus, mediodorsal	438
		ACC	Anterior Cingulate Cortex	2619
		aINS (L, R)	Agranular Insular Cortex	412
		AMY (L, R)	Amygdala	645
		HIP (L, R)	Hippocampus	1043
		pHIP (L, R)	Parahippocampus	476
	Others	BS	Brain Stem	4876
		Pul (L, R)	Thalamus, pulvinar	729
		Cd (L, R)	Caudate	1050
		Pa (L, R)	Pallidum	402
		NAcc (L, R)	Nucleus Accumbens	276
		Pu (L, R)	Putamen	1141
		FO (L, R)	Frontal Operculum Cortex	294
		IFG_oper (L, R)	Inferior Frontal Gyrus, pars opercularis	844
		OFC (L, R)	Orbito Frontal Cortex	1040
		SMA (L, R)	Supplementary Motor Area	810
		M1 (L, R)	Primary motor cortex	1755
		SMG (L, R)	Supramarginal Gyrus	938
		SPL (L, R)	Superior Parietal Lobule	1343
		PO (L, R)	Parietal Operculum Cortex	230
		HG (L, R)	Heschl's Gyrus	554
		dINS (L, R)	Dysgranular insular cortex	435
		PP (L, R)	Planum polare	64
Pain matrix/ Default mode network		mPFC	Medial prefrontal cortex	1438
Default mode network		PCC	Posterior cingulate cortex	2325

Selected brain regions for region of interest (ROI) analysis of the rs-fMRI data. We selected ROIs associated with the perception of the pain (pain matrix) and default mode network, determined from diffusion tensor image-based atlas template of the rhesus macaque [5].

Supplementary references

1. Nagasaka, K., Takashima, I., Matsuda, K. & Higo, N. Late-onset hypersensitivity after a lesion in the ventral posterolateral nucleus of the thalamus: A macaque model of central post-stroke pain. *Scientific Reports* **7**, 10316 (2017).
2. Hosomi, K. *et al.* Daily repetitive transcranial magnetic stimulation of primary motor cortex for neuropathic pain: a randomized, multicenter, double-blind, crossover, sham-controlled trial. *Pain* **154**, 1065–1072 (2013).
3. Hosomi, K. *et al.* Cortical excitability changes after high-frequency repetitive transcranial magnetic stimulation for central poststroke pain. *Pain* **154**, 1352–1357 (2013).
4. Hosomi, K. *et al.* A randomized controlled trial of 5 daily sessions and continuous trial of 4 weekly sessions of repetitive transcranial magnetic stimulation for neuropathic pain. *Pain* **161**, 351–360 (2020).
5. Calabrese, E. *et al.* A diffusion tensor MRI atlas of the postmortem rhesus macaque brain. *NeuroImage* **117**, 408–416 (2015).
